# Health Anxiety in Medical Students: A Hidden Challenge in Medical Education

**DOI:** 10.1192/j.eurpsy.2025.1016

**Published:** 2025-08-26

**Authors:** N. Boussaid, R. Feki, I. Ben Ayed, N. Smaoui, I. Gassara, N. Charfi, J. Ben Thabet, M. Maalej, M. Maalej, S. Omri, L. Zouari

**Affiliations:** 1 Psychiatry C, Hedi Chaker university hospital center, Sfax, Tunisia

## Abstract

**Introduction:**

It is well-established that medical students often experience health-related anxiety, a phenomenon commonly referred to as “medical student syndrome” in the literature. This condition is believed to arise from exposure to life-threatening diseases during medical training.

Health anxiety is characterized by excessive worry about having a serious illness, often leading to heightened distress and maladaptive health-related behaviors.

**Objectives:**

The aim of this study was to explore and compare health anxiety levels between preclinical and clinical medical students.

**Methods:**

A cross-sectional, descriptive, and analytical study was conducted at the Sfax Medical School from March to June 2024. Medical students were invited to voluntarily complete a self-administered questionnaire, which collected socio-demographic data, lifestyle-related factors (such as substance use, physical activity, and medical history), and the Short Health Anxiety Inventory (HAI-18). The HAI-18 assesses the frequency and intensity of health-related worries and behaviors experienced over the past six months. The total score ranges from 0 to 54 and includes two subscales: the Health Anxiety subscale (items 1–14, range 0–42) and the Negative Consequences of Illness subscale (items 15–18, range 0–12). A score of 18 or higher on the Health Anxiety subscale indicates significant health anxiety.

**Results:**

A total of 285 students participated in the study, with a predominance of females (73.7%).

The mean age was 21.96 ± 2.05 years. Preclinical students constituted 31.9% of the sample, while clinical students comprised 68.1%. Most participants (91.9%) resided in urban areas, and 82.1% reported a moderate socioeconomic status. Additionally, 69.5% of participants were living with their families. A medical history was reported by 21.8% of the participants, while 17.2% had a history of psychiatric difficulties. Among these students, 5% had been hospitalized for a serious illness, for a prolonged duration or on multiple occasions.

Health-related anxiety was observed in 24.6% of participants. It was significantly associated with female gender (p = 0.045), a history of psychiatric difficulties (p = 0.004), and being a clinical medical student (p = 0.04). These factors were identified as key predictors of increased health-related anxiety levels.

**Conclusions:**

Female gender, a history of psychiatric difficulties, and clinical medical training were identified as significant risk factors for health-related anxiety among medical students. These findings suggest the need for targeted interventions to address and alleviate anxiety in these high-risk groups, potentially improving both mental health and academic performance within medical training environments.

**Disclosure of Interest:**

None Declared

